# An Incidentally Detected Synthetic Fiber Embedded in the Corneal Stroma: A Report of a Rare Case

**DOI:** 10.7759/cureus.97737

**Published:** 2025-11-25

**Authors:** Tatsuya Mimura, Yui Nishijima, Daisuke Hasegawa, Satoko Kujiraoka, Naoyuki Matsumoto

**Affiliations:** 1 Ophthalmology, Teikyo University School of Medicine, Tokyo, JPN; 2 Department of Ophthalmology, Tsurumi University Dental Hospital, Yokohama, JPN; 3 Department of Ophthalmology, Tsurumi University School of Dental Medicine, Yokohama, JPN; 4 Department of Diagnostic Pathology, Tsurumi University Dental Hospital, Yokohama, JPN; 5 Department of Pathology, Tsurumi University School of Dental Medicine, Yokohama, JPN

**Keywords:** asymptomatic lesion, corneal foreign body, histopathology, intrastromal opacity, non-biological material, ocular surface, slit-lamp examination, synthetic fiber

## Abstract

Corneal foreign bodies (CFBs) are typically caused by the penetration of external materials such as metallic particles, concrete fragments, or plant matter into the corneal tissue and are usually accompanied by intense pain or a foreign-body sensation. We report an extremely rare case of an intrastromal CFB composed of synthetic fiber, which was discovered incidentally in an asymptomatic patient and confirmed histopathologically. A 54-year-old woman presented with bilateral visual impairment due to cataract. Slit-lamp examination revealed bilateral cataracts and a curved, white, linear foreign body approximately 1 mm in length embedded within the corneal stroma of the right eye. The patient had no history of ocular trauma or surgery. Review of anterior segment photographs taken five years earlier for dry eye evaluation demonstrated an identical finding in the same location, indicating that the lesion had been present for years without symptoms. The foreign body was removed under slit-lamp visualization using a 27-gauge needle and forceps. The extracted material was a slender filament lacking cellular components, consistent with a non-biological synthetic fiber. Following removal, the corneal opacity improved. A review of the literature identified reports of conjunctival granulomas or chronic inflammatory reactions caused by synthetic or cotton fibers, as well as intraocular penetration of fibers following ocular surgery or trauma; however, all previously reported cases were symptomatic. Synthetic fibers, commonly present in clothing and indoor environments, can adhere to the ocular surface and, in rare instances, penetrate into the corneal stroma even in the absence of trauma or symptoms. This case highlights a previously unrecognized route of fiber intrusion and suggests that synthetic fibers should be considered in the differential diagnosis of asymptomatic corneal opacities.

## Introduction

Corneal foreign bodies (CFBs) are among the most common ocular surface injuries encountered in emergency ophthalmic practice. They result from the penetration of exogenous materials, such as metallic particles, dust, wooden or plant fragments, and contact lens fragments, into the cornea [1]. Patients with acute CFBs typically present with severe ocular pain, foreign-body sensation, tearing, photophobia, and conjunctival hyperemia. Indeed, CFBs represent one of the leading causes of emergency eye consultations, second only to corneal abrasions, and their prompt management is crucial for the preservation of visual function [2].

The clinical presentation and prognosis of CFBs vary depending on several factors, including the type of material, depth of penetration, duration of retention, and associated tissue reactivity [2,3]. Metallic fragments often induce rust-ring formation and secondary inflammation, while organic materials such as plant matter or wood increase the risk of infection [2,3].

Asymptomatic intrastromal foreign bodies, particularly synthetic fibers, have rarely been documented, and available reports suggest that deeply embedded materials may remain clinically silent [3,4]. However, clearly confirmed cases are lacking. Because pain-sensitive nerve fibers are abundant in the superficial corneal layers but sparse in the deeper stroma, deep stromal foreign bodies may remain asymptomatic

In other ocular structures, such as the anterior chamber or crystalline lens, asymptomatic foreign bodies have occasionally been reported, and a localized tissue encapsulation or “tolerance mechanism” has been proposed to explain such silent retention [5,6].

Herein, we report a unique case of an incidentally discovered, asymptomatic intrastromal white filamentous foreign body, histopathologically identified as a synthetic fiber. We describe the clinical course, surgical removal, and histopathological findings, and we further review the literature to explore possible mechanisms of intrastromal fiber penetration and the clinical implications of asymptomatic CFBs.

## Case presentation

Methods

A single case report was prepared, accompanied by a literature review to identify similar published cases. A comprehensive search of the MEDLINE database was conducted using the keywords “foreign body”, “fiber”, and “cornea” or “corneal”.

This study was based on anonymized clinical information obtained during routine ophthalmic care and was therefore exempt from institutional ethical review. The investigation adhered to the tenets of the Declaration of Helsinki (revised in 2013) [7] and relevant ethical guidelines for clinical research and case reporting.

Written and/or verbal informed consent was obtained from the patient after a full explanation of the nature and purpose of this report, including the use of clinical images for academic publication.

Results

Case Presentation

A 49-year-old woman presented with a one-year history of gradual visual decline and dry eye symptoms. Her medical history was unremarkable, and she had no prior ocular surgery. At her initial visit, the best-corrected visual acuity (BCVA) was (20/28) in the right eye and (20/16) in the left eye. Intraocular pressure was 18 mmHg in both eyes. She reported no foreign body sensation, redness, or discharge. Slit-lamp examination revealed bilateral mild corneal erosion and cataracts, which were attributed to the cause of her visual disturbance. Neither the patient nor the examining physician noticed any corneal opacity in the right eye at that time. Sodium hyaluronate 0.1% eye drops (four times daily, both eyes) were prescribed, but the patient did not return for follow-up.

**Figure 1:**
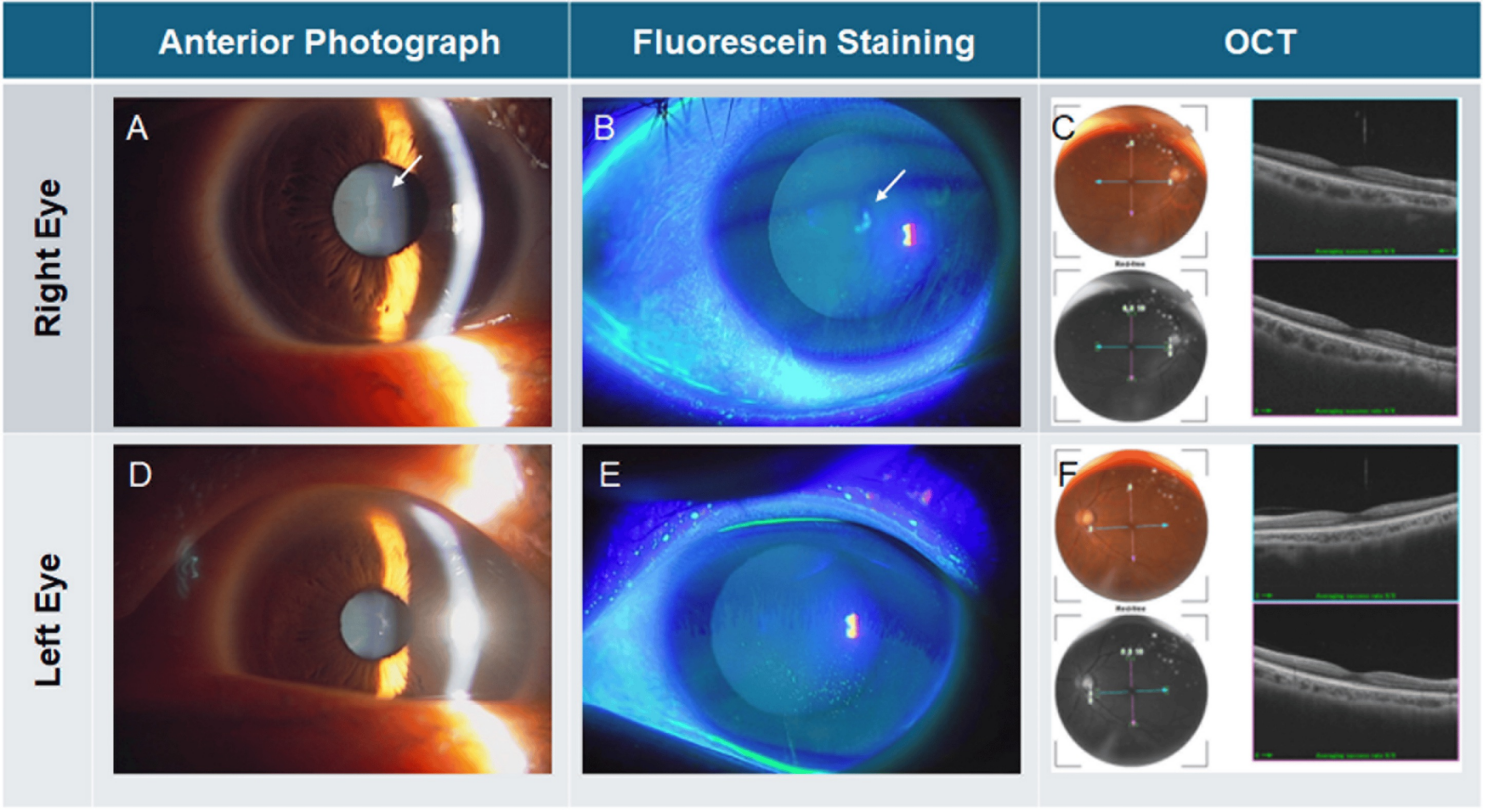
Anterior segment and optical coherence tomography (OCT) images of both eyes at the initial presentation (age 49). (A–C) right eye; (D–F) left eye

Five years later, at the age of 54, she revisited our clinic complaining of further visual decline in both eyes. The previously recorded anterior segment photographs and optical coherence tomography (OCT) images were reviewed to reassess the retinal condition (Figure 1A-1F). Bilateral corneal erosion and cataracts were observed (Figure 1A, 1D). A faint stromal opacity was observed in the right cornea, but no distinct foreign material was visible under routine illumination (Figure 1A). Fluorescein staining of the affected area was negative; however, under cobalt-blue illumination, a fine, curved, white linear structure became apparent (Figure 1B). OCT imaging showed no macular or optic nerve abnormalities in either eye (Figure 1C, 1F).

**Figure 2:**
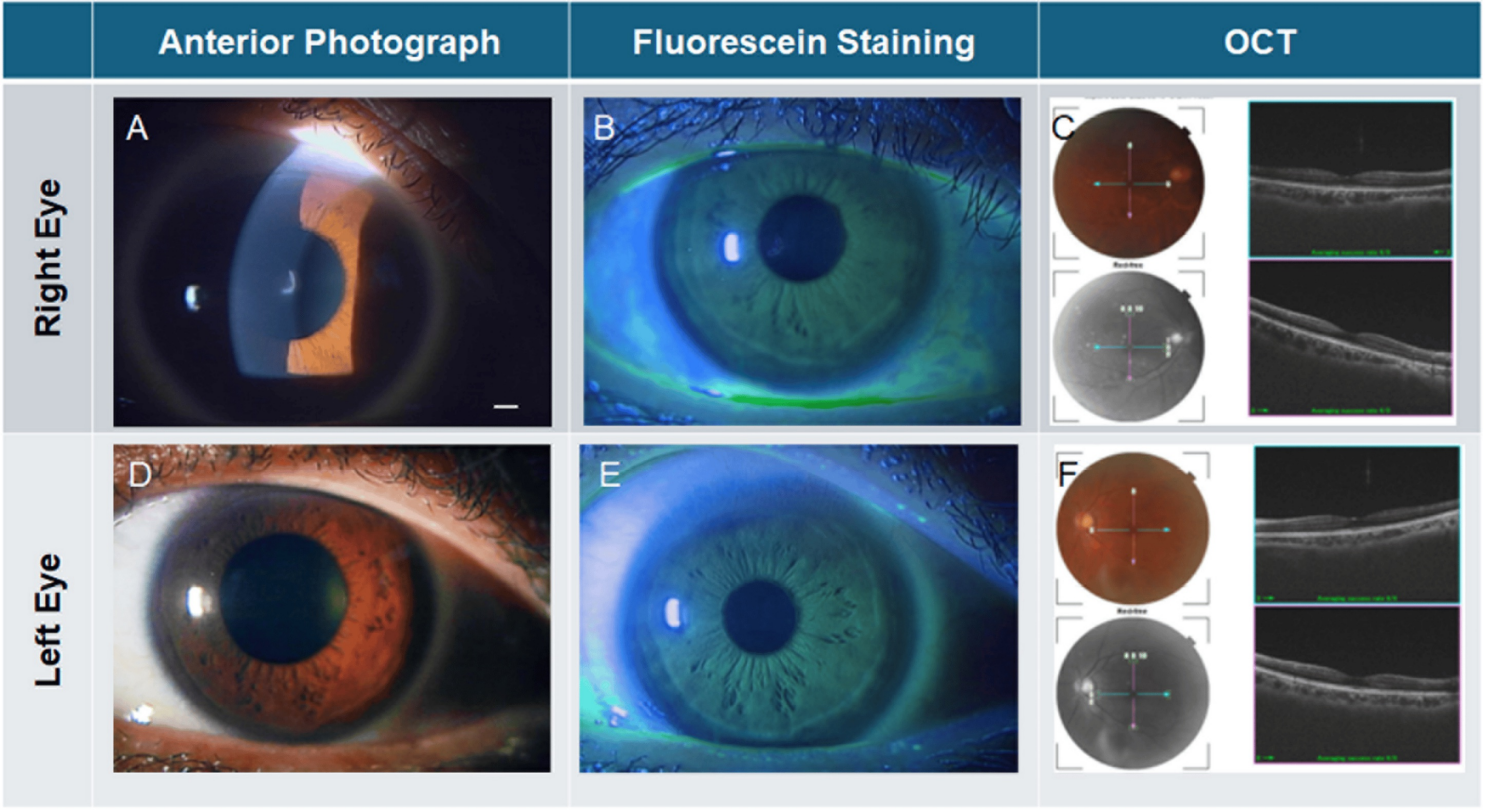
Anterior segment and fundus photographs at follow-up (age 54). (A–C) right eye; (D–F) left eye

Anterior segment and fundus photographs obtained at the current visit (age 54) are shown in Figure 2A-2F. At this visit, BCVA was (20/22) in the right eye and (20/20) in the left eye. Intraocular pressure measured 16 mmHg in the right eye and 14 mmHg in the left. Slit-lamp examination confirmed bilateral cataracts (Figure 2A, 2D) and revealed a curved, whitish, linear structure approximately 1 mm in length embedded within the mid-stroma of the right cornea (Figure 2A), corresponding to the area previously noted under the blue filter. The fundus was unremarkable in both eyes (Figure 2C, 2F).

**Figure 3:**
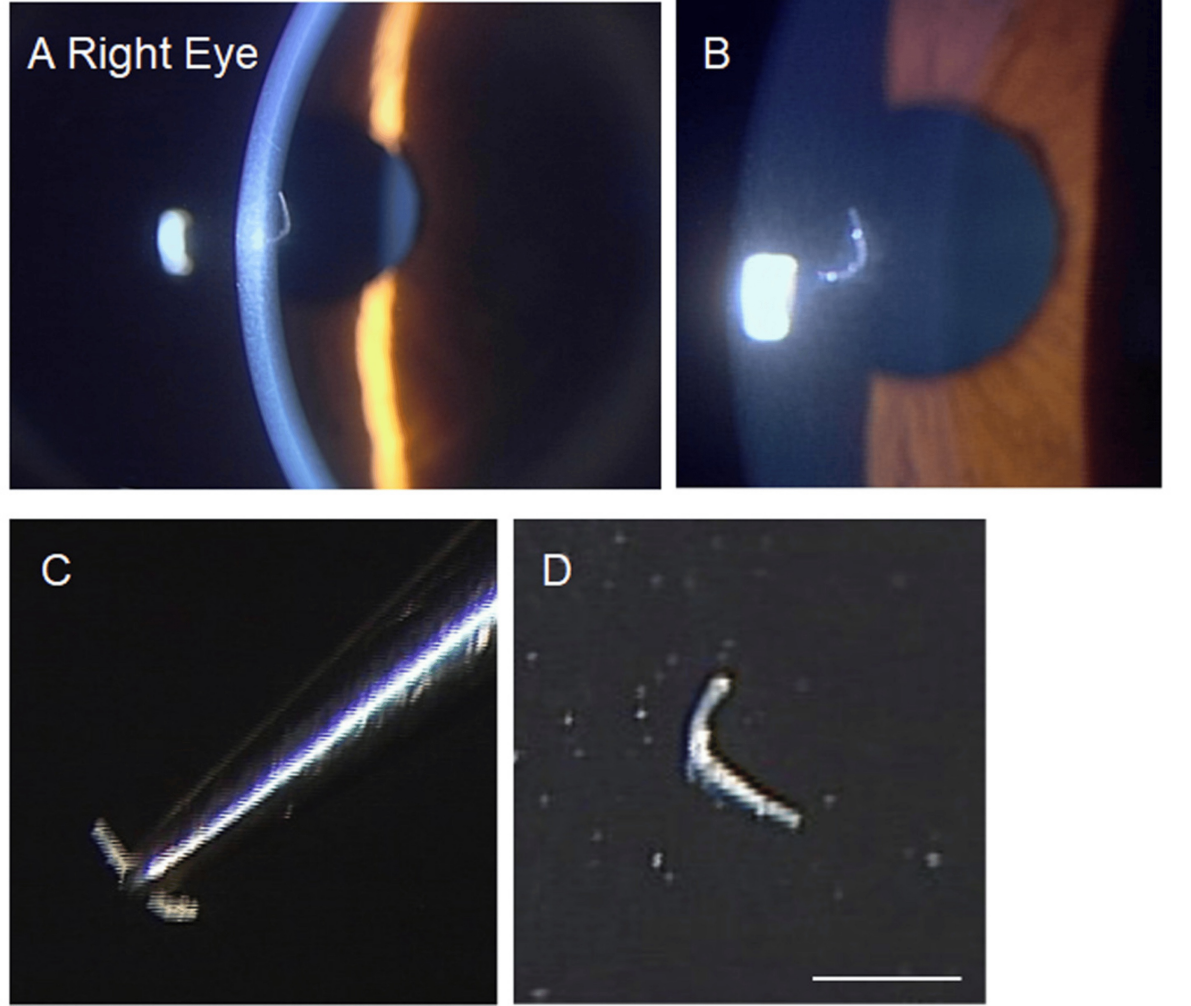
Intrastromal corneal foreign body in the right eye (age 54). (A, B) Magnified slit-lamp photographs of the stromal opacity. (C, D) Photographs of the excised foreign body. Scale bar = 1 mm.

**Figure 4:**
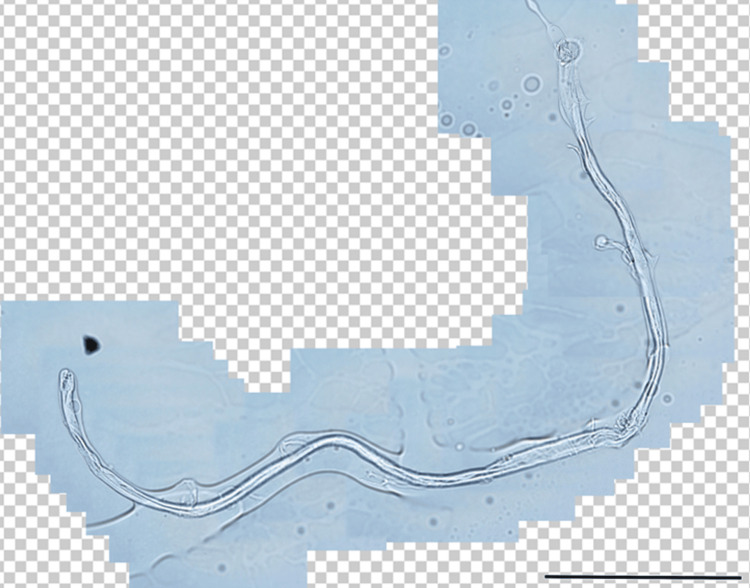
Histopathological images of the extracted foreign body. Microscopic view at 40 × magnification. Scale bar = 250 µm.

A stromal nematode remnant or plant fragment was initially suspected. Under slit-lamp guidance, the lesion was excised using a 27-gauge needle and fine forceps. The removed specimen was a thin, elongated fiber (Figure 3). Histopathologic analysis revealed an acellular structure consistent with a synthetic (man-made) fiber, excluding plant or animal origin (Figure 4). When straightened, its length measured 1.39 mm.

**Figure 5:**
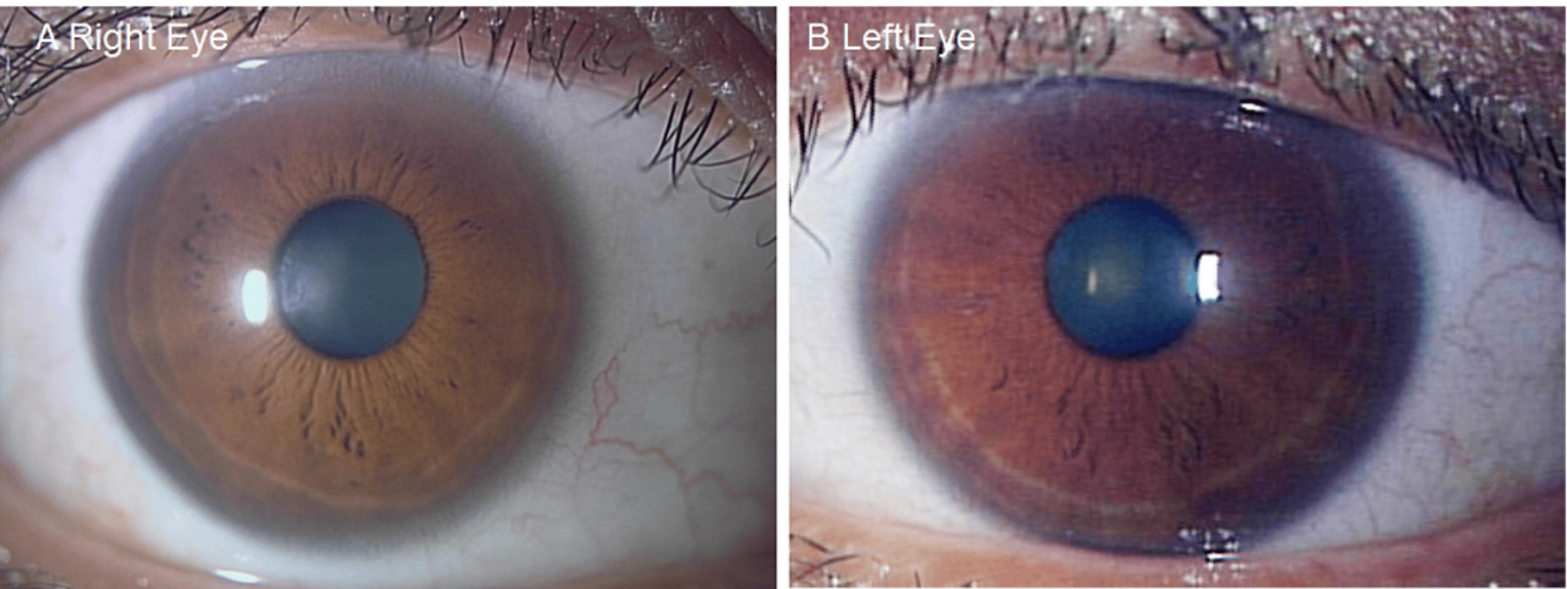
Anterior segment photographs one week after removal of the right corneal foreign body. (A) right eye; (B) left eye. Corneal opacity in the right eye is reduced, and no epithelial erosions are observed.

At one week postoperatively, the corneal opacity had resolved completely, and the corneal clarity was comparable to that of the fellow eye (Figure 5A, 5B).

Based on the available evidence, the fiber had likely been embedded in the corneal stroma for at least five years. Its transparency at the initial visit likely prevented recognition. The fiber exhibited autofluorescence under a cobalt-blue filter, suggesting early stromal penetration. The absence of discomfort, inflammation, or epithelial disruption throughout the five-year period indicates a non-reactive, inert behavior of the foreign material. The gradual whitening of the fiber may have resulted from aging-related material degradation, leading to its incidental detection at the latest visit. This case, therefore, represents an extremely rare instance of an asymptomatic, long-standing intrastromal synthetic fiber.

Long-term follow-up data are not yet available, and the patient continues to be monitored to assess future clinical changes

## Discussion

Literature review, comparison with previous reports, and clinical implications

A comprehensive MEDLINE search using the terms “foreign body”, “fiber”, and “cornea” or “corneal” identified 27 and 31 articles, respectively. After screening, only five case reports describing intraocular or intrastromal fibers were found: an animal-derived fiber penetrating from the sclera into the vitreous cavity [8]; a fiber entrapped within a radial keratotomy incision [9]; a cotton fiber retained at the LASIK flap interface [10]; a cosmetic hair-extension fiber penetrating the cornea and anterior chamber and causing anterior uveitis [11]; and a caterpillar seta entering the vitreous cavity and inducing panuveitis [12]. These cases, summarized in Table 1, involved fibers located within surgical wounds, the anterior chamber, or the vitreous cavity, and all were associated with symptoms such as inflammation, ocular discomfort, or visual disturbances. Reports of conjunctival foreign bodies, including synthetic fibers from toys producing granulomas or chronic irritation, were likewise symptomatic [13,14].

**Table 1:** Representative cases of ocular foreign bodies composed of fibrous material. F = female, M = male

Case	Age/sex	Symptoms / clinical findings	Type of fiber	Location	Treatment	Reference
1	11/F	Ocular pain, vitreous opacity	Animal-derived fiber	Perforated sclera protruding into the vitreous	Vitrectomy	*Cesk Slov Oftalmol, *2001 [1]
2	41/F	Foreign body sensation	Cotton fiber	Radial keratotomy incision	Observation	*J Cataract Refract Surg*, 2005 [8]
3	26/F	Subconjunctival hemorrhage	Cotton fiber	LASIK flap	Observation	*Middle East Afr J Ophthalmol*, 2012 [9]
4	73/M	Anterior uveitis	Hair growth cosmetic fiber	Anterior chamber	Topical therapy	*Am J Ophthalmol Case Rep*, 2020 [10]
5	-/F	Panuveitis, foreign body sensation	Caterpillar setae	Vitreous	Vitrectomy	*Eur J Ophthalmol,* 2024 [11]
6	54/F	Asymptomatic	Cotton fiber	Corneal stroma	Surgical removal	Present case

In addition to naturally occurring or surgical fibers, inadvertent introduction of cotton fibers into the anterior chamber during cataract surgery has been reported in 19 [15] and 14 cases [16]. Although iatrogenic, these reports demonstrate that inert fibers may persist for extended periods, suggesting a degree of ocular tolerance. Other studies have documented asymptomatic foreign bodies in the anterior chamber or crystalline lens [5,6], further indicating that ocular tissues can encapsulate and isolate certain materials without triggering inflammation.

In contrast to all previously published cases, the present report documents an entirely asymptomatic intrastromal synthetic fiber that remained clinically silent for at least five years. This expands the known clinical spectrum of CFBs by demonstrating that transparent, inert materials embedded in the deep stroma may remain undetected under routine slit-lamp examination. This clinical observation carries practical implications: transparent fibers may be missed without targeted examination techniques, and cobalt-blue illumination or fluorescein staining may significantly enhance detection. Furthermore, recognition of such asymptomatic fibers is important in differentiating benign intrastromal materials from infectious, inflammatory, or parasitic lesions and may guide safe surgical planning when incidental corneal opacities are encountered

Case summary

We report an exceptionally rare case of a synthetic fiber-like linear foreign body that remained asymptomatic within the right corneal stroma for at least five years, without causing extrusion, inflammation, or persistent epithelial erosion. The linear structure observed under cobalt-blue illumination at the follow-up visit was subsequently surgically removed and histologically confirmed as a synthetic fiber, highlighting the uniqueness of this case. Postoperative corneal healing was uneventful, and there was no long-term adverse effect on visual function or corneal transparency. A review of the literature revealed few, if any, similar reports of asymptomatic, long-standing intrastromal foreign bodies. This case provides complementary clinical insight to the conventional understanding of CFBs, which typically emphasizes acute, symptomatic presentations.

Potential mechanisms of entry and long-term retention

The precise entry route of the foreign body in this case remains unclear, but several mechanisms are plausible. Microscopic defects in the corneal epithelium, occurring during routine daily activities, may have allowed a transparent or semi-transparent fiber to penetrate into the stroma. Minor ocular surface trauma is common and may go unnoticed by patients. Initially, the foreign body’s transparency likely resulted in minimal optical contrast, rendering it clinically undetectable under standard illumination. Over time, the corneal tissue may have encapsulated the fiber, minimizing tissue reaction and establishing a state of long-term tolerance. Ocular tissues are known to isolate foreign materials through encapsulation, fibrous coating, and suppression of inflammatory responses, as supported by previous reports of asymptomatic anterior chamber metal foreign bodies persisting for extended periods [6].

In addition, the inert nature of the synthetic fiber, lacking chemical reactivity or irritative properties, likely contributed to minimal host response and long-term stromal retention. Although anterior segment OCT (AS-OCT) was not performed in this case, recent studies demonstrate its utility in visualizing CFB depth and reflectivity, aiding risk assessment prior to removal [17]. Such imaging may enhance procedural safety in similar cases.

Reasons for the asymptomatic course

Several factors likely contributed to the absence of pain, foreign-body sensation, or conjunctival hyperemia in this patient. In the human cornea, nerve endings, particularly within the subbasal plexus of the epithelium, are densely distributed and primarily responsible for nociception. In contrast, stromal nerves are sparser, making deep stromal stimuli less perceptible [18, 19]. Consequently, a foreign body located in the deep stroma would be less likely to elicit discomfort.

Furthermore, the inert nature of the synthetic fiber likely prevented mechanical or chemical damage to surrounding tissues, reducing the likelihood of inflammation. Encapsulation or low-inflammatory fibrous coating may have stabilized the interface between the foreign body and corneal tissue. Previous reports have described ocular tolerance of foreign bodies, such as metal objects remaining in the anterior chamber asymptomatically for up to 15 years [6]. Taken together, these factors explain the prolonged asymptomatic presence of a transparent fiber in the deep corneal stroma.

Clinical significance: why recognizing asymptomatic fiber matters

This case emphasizes that asymptomatic intrastromal fibers may remain undetected under routine white-light slit-lamp examination, particularly when the material is transparent. Recognition of this entity is clinically important because such fibers may influence surgical planning, be misinterpreted as inflammatory debris, or be revealed only under cobalt-blue illumination. Awareness of this silent presentation may prevent misdiagnosis and facilitate timely and safe removal when indicated.

Limitations and future directions

This report is limited by its single-case design, restricting generalizability. The timing and precise route of entry, as well as the progression of stromal embedding, remain speculative. While histopathological analysis was performed, detailed chemical characterization of the fiber was not undertaken, leaving some uncertainty regarding its exact composition. AS-OCT imaging was not used preoperatively, limiting precise assessment of stromal depth and tissue interface. Additionally, long-term postoperative monitoring of corneal topography, refractive changes, and subtle visual function was not performed.

Future studies should accumulate additional cases, incorporating AS-OCT imaging and material analysis to establish systematic insights. Basic research into corneal tolerance mechanisms, encapsulation processes, and ocular immune privilege may further elucidate the biological basis for asymptomatic foreign body retention.

## Conclusions

This case highlights a rare instance of a long-standing, asymptomatic synthetic fiber embedded in the corneal stroma. Transparent intrastromal fibers may remain undetected during routine examination, and cobalt-blue illumination or fluorescein staining can improve detection. Awareness of this silent presentation broadens the understanding of corneal foreign bodies and may aid clinicians in evaluating subtle stromal opacities.
